# Autistic traits and positive psychotic experiences modulate the association of psychopathic tendencies with theory of mind in opposite directions

**DOI:** 10.1038/s41598-017-06995-2

**Published:** 2017-07-25

**Authors:** Steven M. Gillespie, Ian J. Mitchell, Ahmad M. Abu-Akel

**Affiliations:** 1 School of Psychology, Newcastle University, Newcastle, NE1 7RU UK; 2 School of Psychology, University of Birmingham, Birmingham, B15 2TT UK; 3 Institute of Psychology, University of Lausanne, Lausanne, 1015 Switzerland

## Abstract

Various clinical disorders, including psychopathy, and autism and schizophrenia spectrum disorders, have been linked with impairments in Theory of Mind (ToM). However, although these conditions can co-occur in the same individual, the effect of their inter-play on ToM abilities has not been investigated. Here we assessed ToM abilities in 55 healthy adults while performing a naturalistic ToM task, requiring participants to watch a short film and judge the actors’ mental states. The results reveal for the first time that autistic traits and positive psychotic experiences interact with psychopathic tendencies in opposite directions to predict ToM performance—the interaction of psychopathic tendencies with autism traits was associated with a *decrement* in performance, whereas the interaction of psychopathic tendencies and positive psychotic experiences was associated with *improved* performance. These effects were specific to cognitive rather than affective ToM. These results underscore the importance of the simultaneous assessment of these dimensions within clinical settings. Future research in these clinical populations may benefit by taking into account such individual differences.

## Introduction

Theory of mind (ToM), variably referred to as cognitive empathy and mentalizing, represents a core aspect of human social interaction, allowing one to infer the mental states of others^1^. Importantly, these abilities can be reliably dissociated into cognitive and affective components^2^. Cognitive ToM refers to the ability to infer the thoughts, intentions, and beliefs of another, while affective ToM allows one to understand another’s feelings and emotions. Evidence to support this distinction is provided by a series of lesion studies showing that patients with lesions to the ventro-medial prefrontal cortex (vmPFC) were impaired during tasks that require an understanding of another’s emotional state, despite showing preserved cognitive ToM^2–4^. Further, although a similar network of structures is activated during cognitive and affective ToM, affective ToM also recruits additional regions required for the integration of cognitive and affective information^5^. Importantly, affective ToM should not be confused with emotional empathy, otherwise termed affective resonance or the ability to feel what another is feeling^6^. Rather, affective ToM refers to the ability to understand others affective mental states.

Evidence suggests that these two broad components of ToM may be differentially affected by several clinical conditions, including psychopathy^7^, autism spectrum conditions [ASCs]^8^, and schizophrenia spectrum disorders [SSDs]^9, 10^. A dimensional view of psychopathology suggests that subclinical manifestations of these conditions are present in the general population, and can co-occur to varying degrees^11–14^. We would note that although the co-occurrence of ASCs and SSDs may appear controversial, recent evidence suggests that these conditions co-occur at an elevated rate, and several models have been proposed to explain their co-occurrence^13, 15, 16^. Furthermore, subclinical manifestations of these conditions can also explain individual differences in ToM and related abilities^11, 17–20^. Importantly, when measured simultaneously in the general population, the precise pattern of ToM impairment observed in relation to these disorders has been found to differ^17^. However, despite recognition that these conditions can co-occur, the extent to which this co-occurrence can explain individual differences in ToM abilities has remained largely unexplored.

Psychopathy refers to a disorder of personality that is characterized by interpersonal and affective features, including interpersonal charm and a lack of empathy, and lifestyle and antisocial features, including recklessness and impulsivity^21^. Evidence suggests that psychopaths are impaired at identifying the affective rather than cognitive states of others. For example, psychopaths show difficulty judging others emotions based on facial expression information^22^, and based on simulated interpersonal interactions^23^. However, psychopaths may perform at a normal level when asked to judge the affective states of others based on pictures of the eye region^24^. In addition, while psychopathic traits in a forensic sample are associated with impairments in inferring another’s affective state, these traits are unrelated to inferring another’s cognitive state^7^. Impairments in affective^25^, but not cognitive^20^, ToM have also been found in relation to the broader psychopathy phenotype in the general population.

Autistic individuals also show problems in ToM functioning^8^. Autism refers to a neurodevelopmental disorder characterised by social interaction and communication problems, and the presence of restrictive, repetitive behavioural patterns^26^. Unlike individuals with psychopathy, individuals with autism show problems in both the cognitive^27, 28^, and affective domains of ToM^29^. However, it should be noted that the nature of affective ToM impairments in autism has been debated, and it is argued that these may reflect commonly comorbid alexithymia – a condition associated with difficulties in identifying and describing one’s own emotions^30^. It has also been shown that the broader phenotype of autism in the general population, but not that of psychopathy, is associated with cognitive ToM impairments^20^. A similar distinction has also been observed in developmental studies of boys with ASCs or psychopathic tendencies^31^, and it has been shown that both autistic and psychopathic traits are associated with ToM problems in children with conduct problems^32^. Taken together, these findings suggest that psychopathy and autism are associated with differing ToM profiles^33–35^.

Since both autism and psychopathy are associated with ToM problems, it has been argued that a ‘double-hit’ of psychopathic and autistic traits would lead to a more pronounced impairment in socio-cognitive abilities^32^. However, to date, there is a lack of empirical support for this position. In one study of 92 adolescents with ASCs, it was found that although psychopathic tendencies co-occurred at an elevated rate, these tendencies were associated with impaired fear recognition but not impairments in cognitive ToM or cognitive flexibility^14^. In a separate study of 134 children aged three to nine years, unique negative effects were found for both psychopathic tendencies and autistic symptoms on parent-reported levels of ToM^32^. However, the outcome measure failed to distinguish between cognitive and affective aspects of ToM. Crucially, neither study found evidence to support the hypothesis of a more pronounced impairment in ToM among those showing co-occurring autism and psychopathic tendencies.

In contrast, nascent evidence suggests that co-occurring psychopathy and schizophrenia may be associated with an attenuating effect on ToM difficulties. Patients with schizophrenia are primarily characterized by psychotic symptoms, including hallucinations and delusions, and also negative symptoms that include diminished emotional expression and avolition^26^. Although patients with schizophrenia typically show impairments in both the cognitive, and the affective components of ToM^10^, increasing psychopathic tendencies are associated with better ToM abilities in patients with schizophrenia scoring above the cut-off point for a diagnosis of psychopathy^12^. Thus, although both psychopathy and schizophrenia are associated with difficulties in ToM, their co-occurrence was not associated with further decline.

Other studies have examined the performance of patients with schizophrenia with and without a diagnosis of antisocial personality disorder (ASPD) – a disorder that is commonly comorbid with psychopathy – compared with healthy controls. In one study, it was found that patients with schizophrenia in the absence of ASPD showed reduced affective ToM compared with healthy controls^36^. In contrast, two groups of violent offenders with ASPD, both with and without schizophrenia, showed a level of performance that was similar to healthy controls^36^. Thus, the co-occurrence of ASPD and schizophrenia was not associated with a further decline in performance compared with those with schizophrenia alone.

It is argued that measures typically used for the assessment of ToM abilities tap a “narrow” bottom-up view of ToM^37^, and do not tap more cognitively demanding ToM processing. Furthermore, most paradigms assessing ToM in both clinical and sub-clinical populations do not make the distinction between its cognitive and affective components. To account for these significant gaps, we used the Movie for the Assessment of Social Cognition [MASC]^38^, a naturalistic and realistic task that distinguishes between cognitive and affective aspects of ToM. Importantly, this task is sensitive in detecting subtle differences in ToM abilities among healthy adults^19, 39^.

Given that autism and schizophrenia may affect ToM performance differently when co-occurring with psychopathy, the current study aimed to examine the interaction of psychopathic tendencies with autistic traits, and the expression of positive psychotic experiences, in a non-clinical adult sample while performing the MASC. The assessment of positive psychotic experiences is used in this study based on evidence that negative symptoms do not reliably discriminate between ASCs and SSDs^40^. Based on evidence for better ToM abilities in patients with schizophrenia and high psychopathic traits^12^, we predicted that co-occurrence of high psychopathy traits with positive psychotic experiences would be associated with improved cognitive ToM abilities. Although earlier research showed the absence of a ‘double hit’ of co-occurring psychopathy and autism on ToM abilities^32^, we predicted that the use of a more sensitive task that is both naturalistic, and capable of distinguishing between cognitive and affective ToM, would reveal a further decrement in cognitive ToM among individuals presenting with high traits of both autism and psychopathy. In addition, we predicted that psychopathic tendencies would be associated with increasing errors in affective ToM, while autistic traits and positive psychotic experiences would be associated with errors for both cognitive and affective ToM.

## Results

### Statistical analysis

We tested for the effects of autism traits (Autism Quotient; AQ), positive psychotic experiences (Community Assessment of Psychic Experiences positive subscale; CAPEp), primary psychopathic tendencies (Levenson Self Report Psychopathy primary subscale; P-LSRP), and all two-way interactions on total ToM errors using ordinary least squares regression models. All regression models were calculated using *z* standardized predictor values. The rationale for the two-way interactions was motivated by evidence for the co-occurrence of autism and conduct disorders/psychopathic tendencies, on the one hand, and for the co-occurrence of psychosis/schizophrenia and psychopathic tendencies, on the other. While controversial, we also included the interaction of autism and psychosis based on evidence for the co-occurrence of ASCs and SSDs^13, 15, 16^. We did not include the three-way interaction as, to our knowledge, there is no evidence suggesting that psychopathy, autism and psychotic spectrum disorders simultaneously co-occur. Significant interactions were probed with the Johnson-Neyman method using MODPROBE for SPSS^41^. This method provides a ‘high-resolution picture’ of the interaction by estimating the value(s) of one predictor, at which the other predictor has a significant effect on the outcome measure. This is established by identifying the precise value(s) along the continuum of one predictor for which the regression slopes of the other predictor are estimated to be significantly different from zero.

Table 1 summarizes the sample scores on the various questionnaires and theory of mind measures.

**Table 1:** Mean ± SD for all self-report questionnaire measures, the National Adult Reading Test, and the number of errors on the Movie for the Assessment of Social Cognition (N = 55).

Measure		Mean ± SD	Range
Levenson Self Report Psychopathy scale (LSRP)			
Overall Score		49.49 ± 10.48	32–82
Primary traits (P-LSRP)		28.40 ± 8.08	17–56
Secondary traits (S-LSRP)		21.09 ± 4.23	10–30
The Community Assessment of Psychic Experiences (CAPE)- Positive Subscale		25.82 ± 3.91	20–37
The Autism Spectrum Quotient (AQ)		13.78 ± 5.66	5–29
Centre for Epidemiologic Studies Depression Scale – Revised (CESD-R)		16.09 ± 14.84	0–64
The National Adult Reading Test (NART)			
Full Scale IQ		103.78 ± 7.32	91–121
NART Verbal IQ		102.65 ± 6.80	91–118
NART Performance IQ		103.98 ± 6.45	93–121
The Movie for the Assessment of Social Cognition (MASC)			
Total number of Errors		9.71 ± 4.16	2–28
Proportion Cognitive Errors ( = N/28)		0.21 ± 0.11	0.00–0.71
Proportion Affective Errors ( = N/17)		0.23 ± 0.11	0.00–0.47

### Total ToM errors

There were no significant relationships of full scale IQ, verbal IQ, performance IQ, age, CESD-R, or S-LSRP with total ToM errors (all *r* < 0.21 and > −0.12, all *p* > 0.13). In addition, there were no significant correlations between the different predictor variables: P-LSRP, AQ, and CAPEp (all r < 0.22, all *p* > 0.12). There was also no difference in the number of overall ToM errors between male and female participants (*t* = 1.05, df = 53, *p* = 0.30).

The overall regression model was significant *F*(6, 48) = 7.54, *p* < 0.001, Δ*R* ^*2*^ = 0.42). Although parameter estimates showed a significant positive relationship of P-LSRP with total ToM errors (β (SE) = 1.68 (0.47), *t* = 3.60, *p* < 0.001), this effect should be interpreted in light of a positive interaction of P-LSRP with AQ scores (β (SE) = 0.97 (0.42), *t* = 2.30, *p* = 0.026), and a negative interaction with CAPEp scores (β (SE) = −1.71 (0.45), *t* = 3.81, *p* < 0.001). No other main effects or interactions were significant.

**Figure 1:**
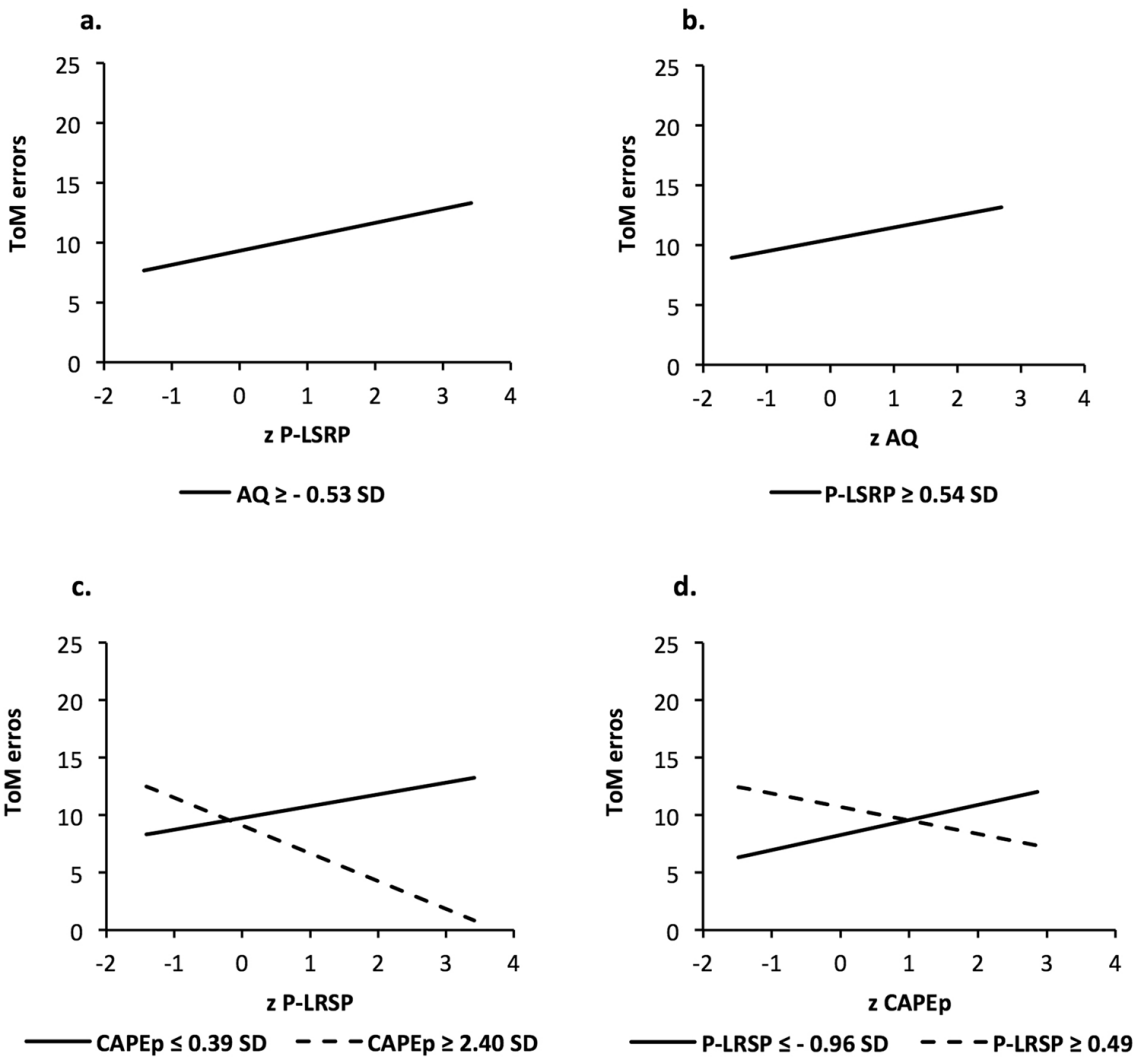
Visualization of the interactive effect of primary psychopathy (P-LSRP) and autism traits (AQ), and of P-LSRP and positive psychotic experiences (CAPEp) on overall theory of mind (ToM) errors. For all panels (**a**–**d**), regression lines represent the significant zone (p ≤ 0.05) of the focal predictor at the specified value of the other predictor. Panels 1a and 1b probe the interaction between the P-LSRP and AQ scores. Panel 1a shows that increasing P-LSRP scores significantly increases ToM errors when AQ scores are equal or more than −0.53 SD from the mean. Panel 1b shows that increasing AQ scores significantly increase ToM errors when P-LSRP scores are equal or more than 0.54 SD from the mean. Panels 1c and 1d probe the interaction between the P-LSRP and CAPEp scores. Panel 1c shows that increasing P-LSRP scores significantly increase ToM errors when CAPEp scores are equal or less than 0.39 SD from the mean, but reduce ToM errors when CAPEp scores are more than 2.40 SD from the mean. Panel 1d shows that increasing CAPEp scores significantly increase ToM errors when P-LSRP scores are equal or less than −0.96 SD from the mean, but reduce ToM Errors when P-LSRP scores are equal or more than 0.49 SD from the mean.

The Johnson-Neyman analysis, presented in Fig. 1a,b, probes the interaction of P-LSRP and AQ. The analysis showed that including the interaction improved the model by 5.65% (*F* = 5.27, *p* = 0.026). As shown in Fig. 1a, the relationship of P-LSRP with the number of ToM errors was positive when AQ scores were at and higher than −0.53 SD from the mean (at AQ = −0.53 SD; β (SE) = 1.17 (0.58), *t* = 2.01, *p* = 0.05, CI_95_ = 0.00–2.34). Conversely, in Fig. 1b, there was a significant positive relationship of AQ with number of ToM errors when the P-LSRP scores were at and higher than 0.54 SD above the mean (at P-LSRP = .54 SD; β (SE) = 1.00 (0.50), *t* = 2.01, *p* = 0.05, CI_95_ = 0.00–2.00) of P-LSRP.

Figure 1c,d probes the interaction of P-LSRP and CAPEp. The analysis showed that including the interaction improved the model by 15.54% (*F* = 14.49, *p* < 0.001). As shown in Fig. 1c, P-LSRP was associated with an increased number of ToM errors when the CAPEp scores were at and lower than 0.39 SD from the mean (at CAPEp = 0.39 SD; β (SE) = 1.02 (0.51), *t* = 2.01, *p* = 0.05, CI_95_ = 0.00–2.04). However, this effect was reversed when the CAPEp scores were at and higher than 2.40 SD from the mean (at CAPE = 2.40 SD; β (SE) = −2.42 (1.20), *t* = −2.01, *p* = .05, CI_95_ = −4.84–0.00). In Fig. 1d, there was a significant positive relationship of CAPEp and number of ToM errors when the P-LSRP scores were at and lower than −0.96 SD from the mean (at P-LSRP = −0.96; β (SE) = 1.31 (0.65), *t* = 2.01, *p* = 0.05, CI_95_ = 0.00–2.62). However, this relationship reversed such that there was a significant negative relationship of CAPEp with number of ToM errors when the P-LSRP scores were at and higher than .49 SD from the mean (at P-LSRP = 0.49 SD; β (SE) = −1.17 (0.58), *t* = 2.01, *p* = 0.05, CI_95_ = −2.34–0.00).

### Cognitive and affective ToM errors

Across the full sample, there was no significant difference in the proportion of cognitive and affective errors on the MASC (*t* = 1.46, *p* = 0.15). The relationships of full scale, verbal, or performance IQ, age, S-LSRP, or CESD-R with cognitive and affective ToM were non-significant (all *r* < 0.19 and >−0.17, all *p* > 0.17). The differences between male and female participants in cognitive (*t* = 0.97, df = 53, *p* = 0.34) and affective (*t* = .86, df = 53, *p* = 0.39) ToM errors were non-significant.

### Proportion of cognitive ToM errors

The overall model for predicting the proportion of cognitive ToM errors was significant *F*(6, 48) = 7.18, *p* < 0.001, Δ*R* ^*2*^ = 0.41). Parameter estimates showed that there was a significant relationship of P-LSRP with the proportion of cognitive ToM errors (β (SE) = 0.04 (0.01), *t* = 2.90, *p* = 0.006). However, this effect should be interpreted in light of a positive interaction of P-LSRP scores with AQ (β (SE) = 0.03 (0.01), *t* = 2.26, *p* = 0.029), and a negative interaction with CAPEp scores (β (SE) = −0.05 (0.01), *t* = 4.08, *p* < 0.001) in predicting the proportion of cognitive ToM errors. No other main effects or interactions were significant.

Probing the interaction of P-LSRP with AQ showed that including the interaction improved the model by 5.60% (*F* = 5.10, *p* = 0.029). We found a significant positive relationship of P-LSRP scores with the proportion of cognitive ToM errors when the AQ scores were at and higher than −.31 SD from the mean (at AQ = −0.31 SD; β (SE) = 0.028 (0.014), *t* = 2.01, *p* = 0.05, CI_95_ = 0.00–0.056). Conversely, the relationship of AQ scores with the proportion of cognitive ToM errors was significantly positive when the P-LSRP scores were at or more than .30 SD from the mean (at P-LSRP = 0.30 SD; β (SE) = 0.025 (0.012), *t* = 2.01, *p* = 0.05, CI_95_ = 0.00–0.05).

Probing the interaction of P-LSRP with CAPEp showed that including the interaction improved the model by 18.3% (*F* = 16.67, *p* < 0.001). We found a significant positive relationship of P-LSRP scores with the proportion of cognitive ToM errors when the CAPEp scores were at and lower than .21 SD from the mean (at CAPEp = 0.21 SD; β (SE) = 0.026 (0.013), *t* = 2.01, *p* = 0.05, CI_95_ = 0.00–0.051). However, this effect was reversed such that there was a significant negative relationship of P-LSRP with the proportion of cognitive errors when CAPEp scores were at and higher than 1.79 SD from the mean (at CAPE = 1.79 SD; β (SE) = −0.051 (0.025), *t* = −2.01, *p* = 0.05, CI_95_ = −.10–0.00). Conversely, there was a positive relationship of CAPEp and cognitive ToM errors when the P-LSRP scores were at and lower than −1.15 SD from the mean (at P-LSRP = −1.15; β (SE) = 0.038 (0.019), *t* = 2.01, *p* = 0.05, CI_95_ = .00–0.075). However, this relationship reversed such that the association between CAPEp and proportion of cognitive ToM errors was negative when the P-LSRP scores were at and higher than .21 SD from the mean (at P-LSRP = 0.21 SD; β (SE) = −0.029 (0.014), *t* = −2.01, *p* = 0.05, CI_95_ = −0.057–0.00).

### Proportion of affective ToM errors

The overall model for predicting the proportion of affective ToM errors was significant *F*(6, 48) = 2.42, *p* = 0.04, Δ*R* ^*2*^ = 0.14). Parameter estimates suggested that the proportion of affective errors significantly increased with increasing P-LSRP scores (β (SE) = 0.04 (0.02), *t* = 2.56, *p* = 0.014). No other main effects or interactions were significant.

## Discussion

In the present study, we examined the interplay between psychopathic tendencies, autism traits, and positive psychotic experiences in predicting performance on the MASC, a naturalistic and realistic test of ToM abilities. We found that primary psychopathic traits were associated with ToM task performance, but that these relationships depended on levels of both autism traits and positive psychotic experiences. In particular, we found that the interaction of psychopathic tendencies and autism traits was associated with a *decrement* in ToM performance, whereas the interaction of psychopathic tendencies with positive psychotic experiences was associated with *improved* ToM performance.

Consistent with our hypothesis, there was a significant interaction of primary psychopathic tendencies with autism traits such that high scores on both measures were associated with a greater overall number of ToM errors. This finding is in contrast to those of an earlier study that failed to find an interaction of psychopathic and autistic traits in a developmental sample^32^. The results reported here are therefore the first to show support for the expected deleterious effects of this ‘double-hit’ of psychopathic tendencies and autism traits on ToM abilities. Conversely, the interaction of primary psychopathic tendencies with positive psychotic experiences showed that increasing scores on both scales was associated with significantly improved overall ToM performance. Notably, these results are consistent with findings suggesting that extreme levels of psychopathic tendencies among patients with schizophrenia was associated with better ToM abilities, compared with patients with lower psychopathic tendencies^12^. The results reported here are the first to show evidence for a similar effect in relation to the broader phenotype of these disorders in a non-clinical sample.

When considering the proportion of cognitive and affective ToM errors, it transpires that the interactions we observed for overall ToM errors were specific to cognitive ToM errors. As for the overall ToM errors, the effect of psychopathic tendencies on cognitive ToM errors depended on the relative expression of autism and psychosis traits such that, on the one hand, increasing psychopathic and autistic traits were associated with *increased* cognitive ToM errors, and on the other hand, increasing psychopathic and psychosis traits were associated with *reduced* cognitive ToM errors. Affective ToM errors were uniquely and positively associated with psychopathic tendencies.

In an attempt to shed light on the mechanism underlying this pattern of results, we make two main observations. First, autism traits and positive psychotic experiences interacted with psychopathic tendencies in opposite directions. This pattern of results is consistent with the diametric model^42^, which posits that ASCs and SSDs have opposing effects on ToM abilities, whereby ASCs are associated with reduced ToM and SSDs with hyper-ToM, a tendency to over-attribute/-generate mental states about others^43^. It has been shown that psychopathy and autism are associated with distinct neurocognitive underpinnings^33, 35, 44^, and that the features associated with these conditions may not overlap^32^. Thus, the combined worsening effect of autistic traits and psychopathic tendencies on ToM performance is conceivable in light of robust evidence associating autism, and its broader phenotype, with impaired cognitive ToM^20, 31, 34^.

The mechanism underlying better cognitive ToM abilities in individuals scoring high on both psychopathic tendencies and positive psychotic experiences is less clear. However, we speculate that if positive psychotic experiences are associated with a greater ability to generate hypotheses about the mental states of others^42, 43^, then this might be advantageous for individuals with co-occurring high psychopathic tendencies. Imaging studies have shown that individuals with clinically elevated levels of psychopathy or ASPD show a greater reliance on cognitive processes, thought to represent a compensatory mechanism in the absence of affective processing^36, 45, 46^. Thus, when combined with a greater number of alternative mental state attributions, it is possible that this cognitive route may prove beneficial for individuals with co-occurring high psychopathic tendencies and psychosis traits. Consistent with this hypothesis, there is evidence to suggest that the co-occurrence of psychopathic tendencies in SSDs may be associated with an increased propensity for interpersonal charm, cunning, and instrumental aggression^12^.

Second, we observe that while psychopathic tendencies had a unique and negative effect on affective ToM errors, its effect on cognitive ToM errors was moderated by the relative expression of autistic traits and psychotic experiences. It has been argued that situations that involve affective ToM entail more self-reflection, and require the integration of affective and cognitive information, compared with situations involving cognitive ToM^6^. Psychopathic tendencies are associated with reduced affective resonance to others emotional expressions, and reduced activation of regions including the amygdala and anterior insula that are involved in emotion and empathy^47^. Hypoactivation of these regions has also been observed in relation to psychopathic tendencies during affective ToM task performance, and may reflect a failure to process emotionally salient cues, including facial expressions and body postures^48^. In contrast, autistic traits, as pointed above, are related to impairments in cognitive ToM, but not in affective resonance or emotional empathy, that is, the ability to feel what another is feeling^31, 33, 44^. Indeed, individuals with autism may resonate with the emotional experience of others to a normal, or even heightened, degree^49, 50^. This distinction between psychopathic tendencies and autism traits likely accounts for the absence of a psychopathy-autism interaction in predicting affective ToM.

Taken together, our findings may have implications for understanding the aggressive behaviours associated with psychopathic tendencies. For example, better ToM abilities may be associated with a more cunning and deceitful interpersonal style in psychopathy, and a greater incidence of premeditated or goal directed aggression^12, 51, 52^. Thus, it may be predicted that the aggressive behaviours of those with elevated psychopathic tendencies and high levels of positive psychotic experiences would be more instrumental compared with those who score relatively more highly on one measure compared with the other. In contrast, adolescents with ASCs and elevated psychopathic traits do not appear to show elevated rates of conduct problems^14^. This finding is interesting and suggests that psychopathy related impairments that are not seen in autism, including in resonating with others emotions and understanding others affective states, may account for increased rates of aggression in relation to psychopathic, but not autistic traits^33, 44^.

Our results need to be interpreted in light of several limitations. The sample recruited in this study was relatively small with the majority being female college students. While we observed no effect of participant sex on ToM task performance in the current study, the extent to which there may be sex differences in the relationships should be investigated further, given previous reports highlighting differential relationships among boys and girls in the relationship of psychopathic traits with empathic abilities^53^. Furthermore, despite recent findings on the relationship of psychopathic tendencies with ToM in patients with schizophrenia^12^, these results are yet to be mirrored in clinical patient samples with extreme levels of autism.

Our study is the first to show that autistic traits and positive psychotic experiences interact with psychopathic tendencies in opposite directions to predict ToM performance. This modulation is specific to cognitive rather than affective errors. We are aware of the debate regarding the relevance of effects associated with attenuated clinical expressions in the healthy population to clinical entities. However, these results underscore the importance of the simultaneous assessments of these dimensions within clinical settings, particularly given the increased recognition of the co-occurrence of these conditions, and the dimensional view of psychopathology. Perhaps the inconsistencies in the literature with regard to the relationship between psychopathic tendencies and ToM abilities reflect individual differences in the expression of autism and psychosis. Future research concerned with socio-cognitive abilities in these clinical populations may benefit by taking into account such individual differences.

## Method

### Participants

A sample of 55 healthy adults (16 males, 39 females), aged between 18 and 37 (*M* (*SD*) = 20.00 ± 2.59) were recruited from the student population at the University of Birmingham, UK, and participated in return for course credit. Participants reported no history of psychiatric illness, epilepsy, neurological disorders, brain injury or alcohol or substance abuse problems. Written informed consent was received from each participant after the nature of the study had been explained. All experiments were carried out in accordance with relevant guidelines and regulations and approved by the University of Birmingham Research Ethics Committee.

## Materials

### The Movie for the Assessment of Social Cognition (MASC)

The MASC^38^ is a 15-minute video film depicting several scenarios revolving around four friends making arrangements to meet for dinner, preparing and eating a meal, and playing a game. The scenarios are played out in different locations between two, three, or all four of the characters, both in person and over the phone. Across these different social situations the characters show misunderstandings, express irony and ambiguous body language, act flirtatiously, and make insulting comments. The task consists of 51 multiple-choice questions of which six were control questions. The critical 45 questions aimed to test the participants’ abilities to infer the thoughts, intentions, feelings, and emotions of the characters. Each question was presented following a short clip from the movie. The total number of correct responses reflects an individual’s general ToM performance. In addition, the MASC can distinguish between cognitive and affective ToM on the basis of questions that ask about knowledge, beliefs and intentions (28 questions), versus feelings and emotions (17 questions). An example cognitive question asks, “What does Michael think Cliff is laughing about?” An example affective question asks, “What is Sandra feeling?”

### Levenson Self Report Psychopathy scale (LSRP)

The LSRP^54^ assesses psychopathic traits in non-institutionalized populations. It contains 26 items measured on a four-point Likert scale. Sixteen items measure ‘primary’ traits, including selfishness and a lack of care for others, and the remaining items tap ‘secondary’ traits, which include proneness to boredom and impulsivity. Internal consistency in the present study was Cronbach’s α = 0.88 for the primary subscale (P-LSRP) and 0.70 for the secondary subscale (S-LSRP). There is no recommended clinical cut-off for the LSRP.

### The Community Assessment of Psychic Experiences (CAPE)

The 42-item CAPE questionnaire^55^ is a reliable measure for the assessment of the expression of psychosis dimensions in clinical and research settings. Positive psychotic experiences were assessed using the CAPE’s 20-item positive subscale (CAPEp). The internal consistency of this subscale in this study is good (Cronbach’s α = 0.74). Although there is not a well-established cut-off score for the CAPEp, a score of 50 has been shown to have some validity^56^.

### The Autism Spectrum Quotient (AQ)

The AQ^29^ consists of 50 items that measure the presence of traits associated with the autistic spectrum within the general population. Each item is given a score of 0 or 1. The AQ’s internal consistency in this study is good (Cronbach’s α = 0.71). A score of 32 has been recommended as a clinical cut-off for the AQ^57^.

### The National Adult Reading Test (NART)

The NART^58^ consists of 50 words of irregular pronunciations. The total number of correct pronunciations is used to predict the full, verbal and performance Wechsler Adult Intelligence Scale (WAIS) IQ^59^.

### Centre for Epidemiologic Studies Depression Scale – Revised (CESD-R)

The CESD-R^60^ is a 20-item self-report scale that closely reflects the DSM-IV criteria for depression, and assesses the individual’s level of depressive symptomatology experienced over the last two weeks. The internal consistency in this study is excellent (Cronbach’s α = 0.95). Although a score equal to or above 16 has been recommended for identifying a person at risk for clinical depression, this value has been criticised for producing too many false positives^61^.

### Data availability

The datasets generated during and/or analysed during the current study are available from the corresponding author on reasonable request.
